# Therapeutic potential of third-generation chimeric antigen receptor T cells targeting B cell maturation antigen for treating multiple myeloma

**DOI:** 10.1007/s10238-024-01347-7

**Published:** 2024-04-29

**Authors:** Punchita Rujirachaivej, Teerapong Siriboonpiputtana, Piriya Luangwattananun, Pornpimon Yuti, Yupanun Wutti-in, Kornkan Choomee, Jatuporn Sujjitjoon, Takol Chareonsirisuthigul, Budsaba Rerkamnuaychoke, Mutita Junking, Pa-thai Yenchitsomanus

**Affiliations:** 1 Graduate Program in Clinical Pathology, Department of Pathology, Faculty of Medicine Ramathibodi Hospital, Mahidol University, Bangkok, Thailand; 2 Department of Pathology, Faculty of Medicine Ramathibodi Hospital, Mahidol University, Bangkok, Thailand; 3 Siriraj Center of Research Excellence for Cancer Immunotherapy (SiCORE-CIT) and Division of Molecular Medicine, Research Department, Faculty of Medicine Siriraj Hospital, Mahidol University, Bangkok, 10700 Thailand; 4 Division of Molecular Medicine, Research Department, Faculty of Medicine Siriraj Hospital, Mahidol University, Bangkok, Thailand; 5 Division of Transfusion Science, Department of Medical Technology, Faculty of Associated Medical Sciences, Chiang Mai University, Chiang Mai, Thailand

**Keywords:** Multiple myeloma, Cancer immunotherapy, B-cell maturation antigen, Chimeric antigen receptor, Anti-BCMA-CAR T cells

## Abstract

Multiple myeloma (MM) is an incurable hematologic malignancy characterized by the rapid proliferation of malignant plasma cells within the bone marrow. Standard therapies often fail due to patient resistance. The US FDA has approved second-generation chimeric antigen receptor (CAR) T cells targeting B-cell maturation antigen (anti-BCMA-CAR2 T cells) for MM treatment. However, achieving enduring clinical responses remains a challenge in CAR T cell therapy. This study developed third-generation T cells with an anti-BCMA CAR (anti-BCMA-CAR3). The CAR incorporated a fully human scFv specific to BCMA, linked to the CD8 hinge region. The design included the CD28 transmembrane domain, two co-stimulatory domains (CD28 and 4-1BB), and the CD3ζ signaling domain (28BBζ). Lentiviral technology generated these modified T cells, which were compared against anti-BCMA-CAR2 T cells for efficacy against cancer. Anti-BCMA-CAR3 T cells exhibited significantly higher cytotoxic activity against BCMA-expressing cells (KMS-12-PE and NCI-H929) compared to anti-BCMA-CAR2 T cells. At an effector-to-target ratio of 10:1, anti-BCMA-CAR3 T cells induced lysis in 75.5 ± 3.8% of NCI-H929 cells, whereas anti-BCMA-CAR2 T cells achieved 56.7 ± 3.4% (*p* = 0.0023). Notably, after twelve days of cultivation, anti-BCMA-CAR3 T cells nearly eradicated BCMA-positive cells (4.1 ± 2.1%), while anti-BCMA-CAR2 T cells allowed 36.8 ± 20.1% to survive. This study highlights the superior efficacy of anti-BCMA-CAR3 T cells against both low and high BCMA-expressing MM cells, surpassing anti-BCMA-CAR2 T cells. These findings suggest potential for advancing anti-BCMA-CAR3 T cells in chimeric antigen receptor T (CAR-T) therapy for relapsed/refractory MM.

**Supplementary Information:**

The online version contains supplementary material available at 10.1007/s10238-024-01347-7.

## Introduction

Multiple myeloma (MM) is a hematologic malignancy characterized by the clonal expansion of malignant plasma cells within the bone marrow [1]. This pathological proliferation gives rise to a considerable global burden in terms of morbidity and mortality. As one of the prominent blood cancers, MM contributes to an incidence rate of 176,404 cases and an alarming mortality rate of 177,077 cases. In the year 2020, the age-standardized incidence rate (ASR) of MM within southeastern Asia was documented at 0.96 (95% UI 0.73–1.27) per 100,000 individuals, while the ASR of mortality stood at 0.82 (95% UI 0.62–1.09) per 100,000 individuals [2]. Despite notable therapeutic advancements, MM remains an incurable disease [3–5], compelling the deployment of a therapeutic amalgamation comprising chemotherapeutic agents, immunomodulatory drugs, proteasome inhibitors, anti-CD38 monoclonal antibodies, and autologous stem cell transplantations [6–8]. Regrettably, a subset of MM patients, approximately 10–20%, especially those harboring high-risk phenotypes, succumb within a span of 2–3 years post diagnosis [6, 9]. Moreover, individuals afflicted with relapsed/refractory MM (RRMM) who encounter a triple-class regimen exhibit pronounced refractoriness (73.8%) alongside a conspicuously diminished overall response rate (ORR) (25.1%) [10]. These unfavorable prognoses underscore the pressing exigency for pioneering therapeutic modalities. T-cell–redirecting interventions, exemplified by chimeric antigen receptor (CAR) T cells, offer a promising avenue to ameliorate outcomes among this cohort of patients.

Chimeric antigen receptor (CAR) T cell-based immunotherapy entails the genetic modification of T cells to express a single-chain variable fragment (scFv) antibody that is highly specific to the tumor antigen present on the surface of target cells. Incorporated within the intracellular domain of the CAR are signaling components capable of activating T cell responses [11]. CAR T cells have exhibited remarkable efficacy in combatting relapsed/refractory B-cell lymphoblastic leukemia (ALL) and non-Hodgkin lymphoma (NHL), as underscored by the approval of anti-CD19-CAR T cells [12, 13]. In the realm of relapsed/refractory multiple myeloma (RRMM), two second-generation anti-BCMA-CAR (anti-BCMA-CAR2) T cell therapies—idecaptagene vicleucel (ide-cel) and ciltacabtagene autoleucel (cilta-cel)—have gained U.S. FDA approval for the treatment of adult patients who have undergone a minimum of > 3 prior therapeutic regimens, including proteasome inhibitors, immunomodulatory drugs, and anti-CD38 monoclonal antibodies [13–16]. Cilta-cel, an autologous CAR T cell therapy equipped with dual BCMA receptors targeting distinct epitopes, showcased remarkable clinical performance with an overall response rate (ORR) reaching 97.9% and a stringent complete response (CR) rate of 82.5% [16]. Conversely, a stringent CR rate of merely 28% was observed in 100 RRMM patients treated with ide-cel, which bears a solitary BCMA binding domain [15]. An encompassing meta-analysis by Roex et al. [17] covering 23 distinct anti-BCMA-CAR T cell products demonstrated a pooled ORR of 80.5% coupled with a CR rate of 44.8%. The reduced CR rates in these investigations may be attributed to an immune response against murine anti-BCMA scFv within the CAR2 T cells. Que et al. [18] conducted clinical trials comparing the outcomes of extramedullary MM (EMM) and non-EMM patients treated with either murine or fully human anti-BCMA-CAR T cell therapy. Intriguingly, non-EMM patients subjected to fully human anti-BCMA-CAR T cells exhibited a CR rate of 81.25%, surpassing those treated with murine anti-BCMA-CAR T cells, where the CR rate was 45% [18]. These results suggest that integrating fully human scFv and intracellular constructs, crucial elements of CAR T cells, has the potential to augment the anti-tumor capabilities of CAR T cells. This avenue warrants additional exploration and holds promise for enhancing therapeutic outcomes in patients with multiple myeloma.

The potency of CAR T cells against tumors hinges on the configuration of their intracellular domains. Notably, the anti-BCMA-CAR2 construct, authorized for use, employs the T cell receptor’s intracellular segment (CD3ζ) along with 4-1BB as co-stimulatory components [13, 15, 19]. However, a subsequent iteration, termed third-generation CAR (CAR3) T cells, has emerged, amalgamating CD28 and 4-1BB co-stimulatory molecules. This integration has yielded heightened T-cell proliferation, accelerated tumor elimination, prolonged persistence, and resistance to relapse [20–22]. Ramos et al. [20] employed CD19-CAR3 T cells in treating 16 individuals with relapsed or refractory non-Hodgkin’s lymphoma, revealing enhanced expansion and prolonged survival compared to CD19-CAR2 T cells. This difference was most pronounced in patients with minimal disease burden, where prior therapy had depleted their normal CD19^+^ B cells. Numerous investigations have corroborated the superiority of CAR3 T cells over CAR2 counterparts, underscoring improved effector functions, in vivo persistence, heightened complete response rates, and only moderate toxicity [20–25]. These collective findings advocate the potential advantage of CAR3 T cells, particularly for high-risk multiple myeloma (MM) and relapsed/refractory MM (RRMM) patients. In this context, our study centers on generating anti-BCMA-CAR3 T cells outfitted with fully human anti-BCMA scFv in the extracellular domain. A comparative evaluation was conducted between anti-BCMA-CAR2 T cells and anti-BCMA-CAR3 T cells, featuring signaling domains of 4-1BB/CD3ζ and CD28/4-1BB/CD3ζ, respectively. Our investigations unequivocally demonstrate that anti-BCMA-CAR3 T cells exhibit significantly heightened effectiveness against BCMA-positive tumors, surpassing the capabilities of anti-BCMA-CAR2 T cells.

## Materials and methods

### Cell culture

Human multiple myeloma (MM) cell lines, namely NCI-H929 and KMS12-PE, were graciously provided by Professor Seiji Okada from the Center for AIDS Research and Graduate School of Medical Sciences, Division of Hematopoiesis, Kumamoto University, Kumamoto, Japan. The K562 cell line, derived from human chronic myelogenous leukemia (CML), was procured from American Type Culture Collection (ATCC) (Cat# CCL-243, RRID: CVCL_0004, Manassas, VA, USA). NCI-H929, KMS12-PE, and K562 cell lines were cultured in Roswell Park Memorial Institute (RPMI)-1640 medium (Gibco; Invitrogen Corporation, Carlsbad, CA, USA) supplemented with 10% heat-inactivated fetal bovine serum (FBS) (Gibco; Invitrogen) and 100 μg/ml of penicillin/streptomycin (Sigma-Aldrich Corporation, St. Louis, MO, USA). Lenti-X™ human embryonic kidney (HEK) 293T cell lines (Takara Bio, Inc., Shiga, Japan) were maintained in Dulbecco’s Modified Eagle Medium (DMEM) (Gibco; Thermo Fisher Scientific, Waltham, MA, USA) containing 10% FBS and 100 μg/ml of penicillin/streptomycin. All cell lines were incubated at 37 °C in a 5% CO_2_ atmosphere.

### Construction of anti-BCMA CARs

The fully human anti-B-cell maturation antigen (BCMA) single-chain variable fragment (scFv) sequence was obtained from an international publication WO 2016/014565 A2 BCMA-10 (EG63-98LB; 139109) and modified by the addition of a Bam-HI site at the 5′ terminus, a Myc tag, and a MreI site at the 3′ terminus. Second-generation CAR (CAR2) and third-generation CAR (CAR3) cDNA sequences were synthesized by Integrated DNA Technologies (Coralville, IA, USA). The fully human anti-BCMA scFv codon-optimized gene was sub-cloned into a self-inactivating (SIN) lentiviral vector (pCDH.EF1α.SIN.WPRE, RRID: Addgene_71708) containing expression cassettes encoding CD8 short hinge, CD8 transmembrane domain, and the 4-1BB/CD3ζ signaling domain (anti-BCMA-CAR2) or CD8 short hinge, CD28 transmembrane domain, and the CD28/4-1BB/CD3ζ signaling domain (anti-BCMA-CAR3) (Fig. 2A). Transgene expression is governed by the elongation factor-1a (EF1α) promoter. To construct the lentiviral vectors, the anti-BCMA scFv DNA fragment was amplified with specific primers, digested with Bam-HI and MreI enzymes overnight at 37 °C, and then purified using the QIAquick PCR Purification kit (Qiagen, Hilden, Germany). The anti-BCMA scFv, CAR2, and CAR3 fragments were ligated using the rapid DNA ligation Kit (New England Biolabs, MA, USA). The correctness of the inserted sequence genes in the vector was verified by Sanger sequencing.

### Lentiviral production

In order to generate lentiviral particles, Lenti-X™ HEK 293T cells were co-transfected with pCDH-EF1α-anti-BCMA-CAR2 or CAR3 plasmid and two packaging plasmids (psPAX2 and pMD2.G) through calcium phosphate precipitation. After 48 and 72 h of transfection, the culture supernatants containing lentiviral particles were collected and filtered through a 0.45 μm membrane to eliminate cell debris. The viral particles were then concentrated by high-speed centrifugation at 20,000 g for 180 min at 4 °C and stored at − 80 °C for subsequent experiments. According to the manufacturer’s instructions, the virus titer was quantified using a qPCR Lentiviral Titration Kit (Applied Biological Materials [ABM], Richmond, BC, Canada).

### T cell isolation and transduction

Peripheral blood mononuclear cells (PBMCs) were obtained from healthy volunteers through density gradient centrifugation in Lymphocyte Separation Medium (Corning, Inc., New York, NY, USA). Subsequently, PBMCs were seeded in a culture dish to enable adherence of undesired monocytes in AIM-V medium (Gibco, Waltham, MA, USA) supplemented with 5% human AB serum (Sigma-Aldrich). Non-adherent cells served as a T cell source and were collected for the analysis of PBMC phenotype. Subsequently, T cells were stimulated with 5 μg/ml Phytohemagglutinin-L (PHA-L) (Roche, Basel, Switzerland), recombinant human interleukin (rhIL)-2 (10 ng/ml), rhIL-7 (5 ng/ml), and rhIL-15 (20 mg/ml) (Immunotools, Friesoythe, Germany) for 72 h, after which they were collected for the analysis of PHA-L activated-T cell phenotype. Following this, PHA-activated T cells underwent transduction with lentiviruses in the presence of 10 μg/ml protamine sulfate (Sigma-Aldrich) and were spinoculated at 1,200 g for 90 min at 32 °C. The transduced T cells were then sustained in a culture medium containing rhIL-2 (10 ng/ml), rhIL-7 (5 ng/ml), and rhIL-15 (20 mg/ml). Flow cytometry was employed at 48- or 72-h post-transduction to assess the expression of anti-BCMA-CAR on transduced T cells, and phenotypes were further determined on day 6 after transduction.

### Flow cytometry

To determine the surface expression of B-cell maturation antigen (BCMA), CD269 (BCMA)-APC antibody (Clone REA315; Miltenyi Biotec, Bergisch Gladbach, Germany) was used to stain the washed cell lines. To evaluate the surface expression of chimeric antigen receptors (CARs) in mammalian cells and assess the transduction efficiency of all generations of anti-BCMA-CAR T cells, anti-c-Myc-FITC (Clone ab1394; Abcam, Cambridge, UK) was employed. The T-cell phenotype was characterized by staining with various antibodies, including anti-CD3-FITC (Clone UCHT-1), anti-CD3-PerCP (Clone UCHT-1), anti-CD4-APC (Clone MEM-241), anti-CD8-APC (Clone UCHT-4), anti-CD16-APC (Clone 3G8), anti-CD19-APC (Clone LT19), anti-45RO-FITC (Clone UCHL1), and CD62L-PE (Clone HI62L), all of which were procured from ImmunoTools GmbH (Friesoythe, Germany), while anti-CD56-PE (Clone AB_2563925) was obtained from BioLegend (CA, USA). The exhaustion marker of T cells was determined by staining with the following antibodies: anti-PD1-PE (Clone EH12.2H7), anti-LAG3-PE (Clone C9B7W), and anti-TIM3-PE (Clone F38-2E2), which were purchased from BioLegend. The cells were incubated with the monoclonal antibodies at 4 °C for 30 min in the dark, followed by two washes and analysis with a BD Accuri™ C6 Plus Flow Cytometer (BD Biosciences, Franklin Lakes, NJ, USA). FlowJo 10.0 software (FlowJo LLC, Ashland, OR USA) was used to analyze the data obtained from the flow cytometer.

### Immunoblot analysis

Immunoblotting was utilized to detect BCMA surface expression in cell lines. The cells were lysed in 1% NP-40 lysis buffer, and the extracted proteins were separated by 12% sodium dodecyl sulfate-polyacrylamide gel electrophoresis (SDS-PAGE) and transferred onto a nitrocellulose membrane. The membrane was blocked using 5% skim milk in Tris-buffered saline and 0.1% Tween-20 (TBST), followed by probing with anti-human BCMA (Clone D-6; Santa Cruz Biotechnology, Dallas, TX, USA) and anti-human GADPH (Clone 0411; Santa Cruz Biotechnology) antibodies. To determine the expression of anti-BCMA-CAR2 or anti-BCMA-CAR3, Lenti-X™ HEK 293T cells were transfected with anti-BCMA-CAR2 or CAR3 using Lipofectamine® 2000 Reagent (Life Technologies Corporation, Carlsbad, CA, USA) for 48 h. The transfected Lenti-X™ HEK293T cells were lysed, and their proteins were separated using SDS-PAGE and then probed with anti-human CD3 zeta (CD3ζ) (clone sc-166435; Santa Cruz Biotechnology Dallas, TX, USA). Subsequently, the membrane was incubated with a secondary antibody conjugated with horseradish peroxidase (HRP) (Invitrogen, City, Country), and the immunoreaction was developed using SuperSignal™ chemiluminescent substrate (Thermo Fisher Scientific, Waltham, MA, USA). The signal generated by the reaction was captured on X-ray film and quantified using ImageJ program (National Institutes of Health, Bethesda, MD, USA).

### Flow cytometry-based cytotoxicity and proliferation assay

The target cells were labeled with 0.75 µM of CellTracker™ CMFDA (5-chloromethylfluorescein diacetate) Dye (Thermo Fisher Scientific). Co-culture assays were carried out by incubating anti-BCMA-CAR T cells and un-transduced T cells (UTD T) with CMFDA-labeled target cells for 12 h at effector to target (E:T) ratios of 1:1, 5:1, and 10:1. Following co-culturing, counting beads (123count™ eBeads Counting Beads; Thermo Fisher Scientific) were added to each sample to enable quantification of the absolute number of target cells by flow cytometry analysis, using the manufacturer's instructions. The percentage of tumor cell cytotoxicity was determined using the formula: [1 − (target cells in each condition/target cells alone at the indicated time)] × 100. To assess the proliferation activity, anti-BCMA-CAR T cells and UTD T cells were labeled with 5 μM carboxyfluorescein succinimidyl ester (CFSE) (Invitrogen) and co-cultured with target cells at E:T ratio of 5:1 in the absence of exogenous cytokines. After 5 days of co-cultivation, CFSE dilution due to cell proliferation was evaluated by gating the lymphocyte population using flow cytometry.

### Cytokine production

Anti-BCMA-CAR T cells and un-transduced T cells (UTD T) were co-cultured with KMS-12-PE cells or NCI-H929 cells at a 5:1 E:T ratio in a serum-free medium for 24 h. Following co-culturing, culture supernatants were collected, centrifuged to remove cell debris, and stored at − 20 °C. The levels of cytokines in the culture supernatants were quantified using the LEGENDplex™ Human CD8/NK cell panel (#741065, BioLegend), which enables simultaneous measurement of 13 human cytokines and proteins, including IL-2, IL-4, IL-6, IL-10, IL-17A, IFN-γ, TNF-α, soluble Fas, soluble FasL, granzyme A, granzyme B, perforin, and granulysin, by utilizing the Cytokine Bead Array (CBA) methodology, in accordance with the manufacturer’s instructions. The samples were analyzed using a CytoFLEX flow cytometer (Becton Dickinson (BD) Biosciences, NJ, USA).

### Stress test

The present study employed a stress test to simulate repetitive antigen exposure in vitro, following established protocols [26]. Briefly, NCI-H929 target cells were co-cultured with anti-BCMA-CAR T cells at an E:T ratio of 1:2 for 72 h, representing cycle one. The resulting cell suspension was then analyzed using flow cytometry to determine the number of residual viable BCMA target cells, T cell proliferation rate, frequency of transduced CAR^+^T cells, and exhaustion profile. Absolute effector cell counts were obtained by using counting beads (123count™ eBeads Counting Beads; Thermo Fisher Scientific), and effector cells were transferred to new wells at the same E:T ratio of 1:2. This process was repeated for a total of three cycles. Residual viable cells were stained with anti-CD138-FITC (Clone B-A38), anti-CD3-APC (Clone UCHT-1), and propidium iodide (PI) (all Immunotools). The proliferation rate was assessed using counting beads, which were mixed into each sample for flow cytometry analysis, and the absolute number of effector cells was calculated according to the manufacturer’s instructions. Fold changes of T cell proliferation were determined by dividing the number of effector cells at each cycle by the number of effector cells at the previous cycle. The frequency of transduced CAR^+^T cells was analyzed by staining with anti-CD3-APC (Clone UCHT-1) and anti-c-Myc-FITC (Clone ab1394; Abcam, Cambridge, UK). The exhaustion profile was determined by co-staining CD3^+^T cells with anti-PD1-PE (Clone EH12.2H7), anti-LAG3-PE (Clone C9B7W), and anti-TIM3-PE (Clone F38-2E2).

### Statistical analysis

Statistical analysis was conducted using GraphPad Prism 9.0 (GraphPad Software, San Diego, CA, USA). The data are expressed as mean ± standard error of the mean (SEM). Unpaired Student’s t-tests were performed to compare two groups, and one-way ANOVA with Tukey’s post-hoc test was used to compare three or more groups. A 2-way repeated-measures ANOVA was utilized for the statistical analysis of the stress test. The significance level was set at *p* < 0.05.

## Results

### Expression of B-cell maturation antigen in multiple myeloma (MM) cell lines

**Fig. 1:**
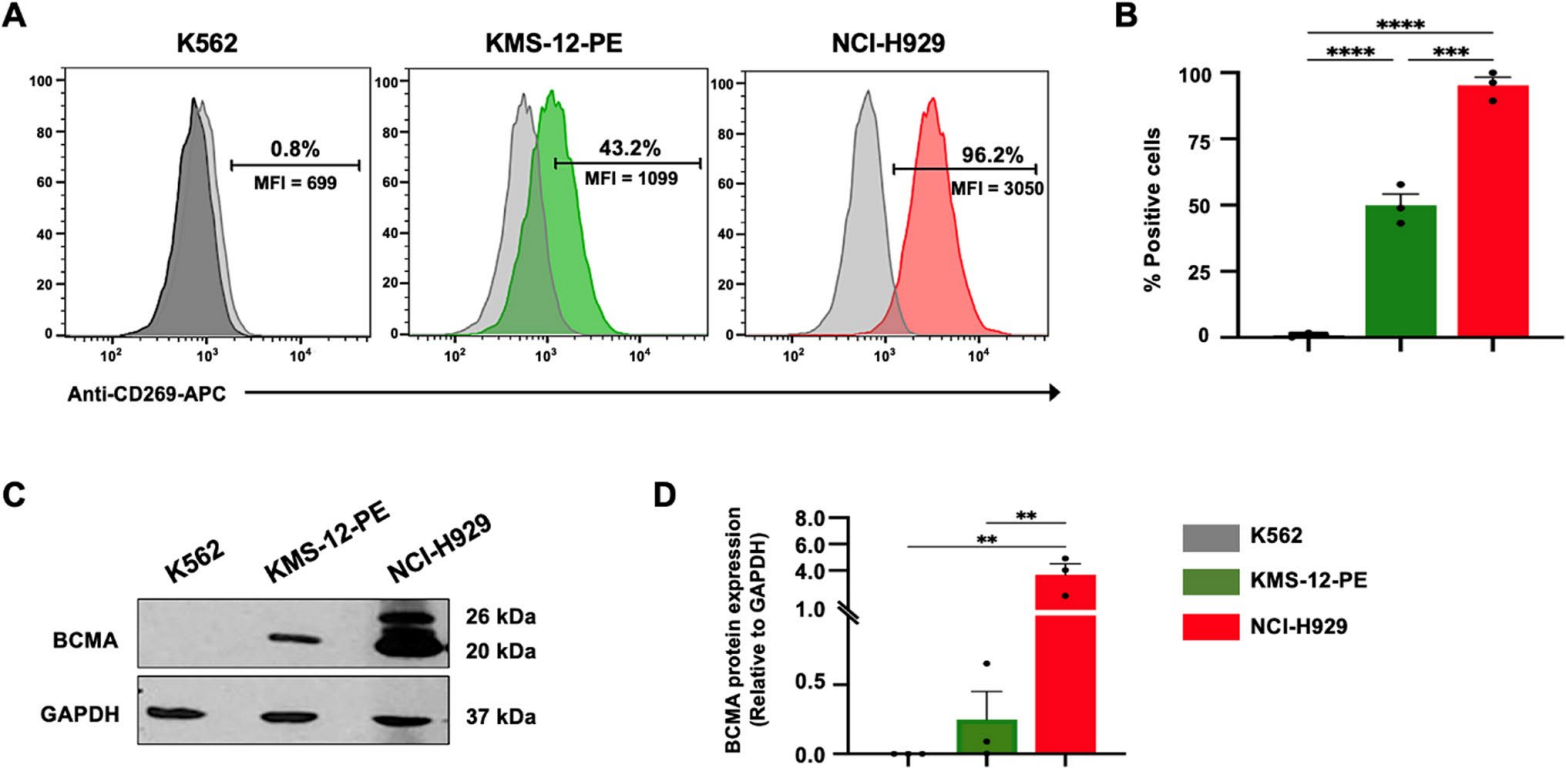
Expression of B-cell maturation antigen (BCMA) in multiple myeloma (MM) cell lines. The expression of BCMA was evaluated in MM cell lines KMS-12-PE and NCI-H929, as well as in non-MM cell line K562, using flow cytometry and immunoblot analysis. **A** Flow cytometry histogram demonstrating surface BCMA expression compared to a matched isotype control (light gray). **B** The percentages of BCMA-positive cells were determined. **C** Immunoblot analysis demonstrated the presence of glycosylated 26 kDa BCMA, 20 kDa BCMA, and 37 kDa glyceraldehyde 3-phosphate dehydrogenase (GAPDH) as loading control. **D** Densitometry analysis was performed to quantify the relative BCMA and GAPDH bands on SDS-PAGE using ImageJ software. The data were obtained from 3 independent experiments, and the results are expressed as the mean ± standard error of the mean (SEM) (N = 3). Statistical significance was determined using one-way analysis of variance (ANOVA) with Tukey's post-hoc test (*p < 0.05, **p < 0.01, ***p < 0.001, ****p < 0.0001) (color figure online)

The present study employed flow cytometry and immunoblot analysis to determine the expression of B-cell maturation antigen (BCMA) in multiple myeloma (MM) cell lines KMS-12-PE and NCI-H929. A CD269 (BCMA)-APC antibody and anti-human BCMA were used for flow cytometry and immunoblot analysis. Human chronic myelogenous leukemia (CML) cell line K562 was used as BCMA low or negative expression cell line. Flow cytometric analysis revealed surface expression of BCMA in MM cell lines KMS-12-PE and NCI-H929. Conversely, K562 cells did not show any detectable surface BCMA expression (0.8 ± 0.5%) (Fig. 1A, B). The MM cell lines KMS-12-PE and NCI-H929 expressed surface BCMA at 50 ± 4.2% (*p* < 0.0001) and 95.2 ± 3.1% (*p* < 0.0001), respectively, in contrast to K562 cells (Fig. 1A, B). Furthermore, the results of the immunoblot analysis were consistent with the surface expression of BCMA detected by flow cytometry. The expression of BCMA in KMS-12-PE and NCI-H929 was significantly higher than in K562 cells (*p* = 0.0047 and *p* = 0.0067, respectively) (Fig. 1C, D).

### Anti-BCMA-CAR construction and expression in Lenti-X™ HEK293T

**Fig. 2:**
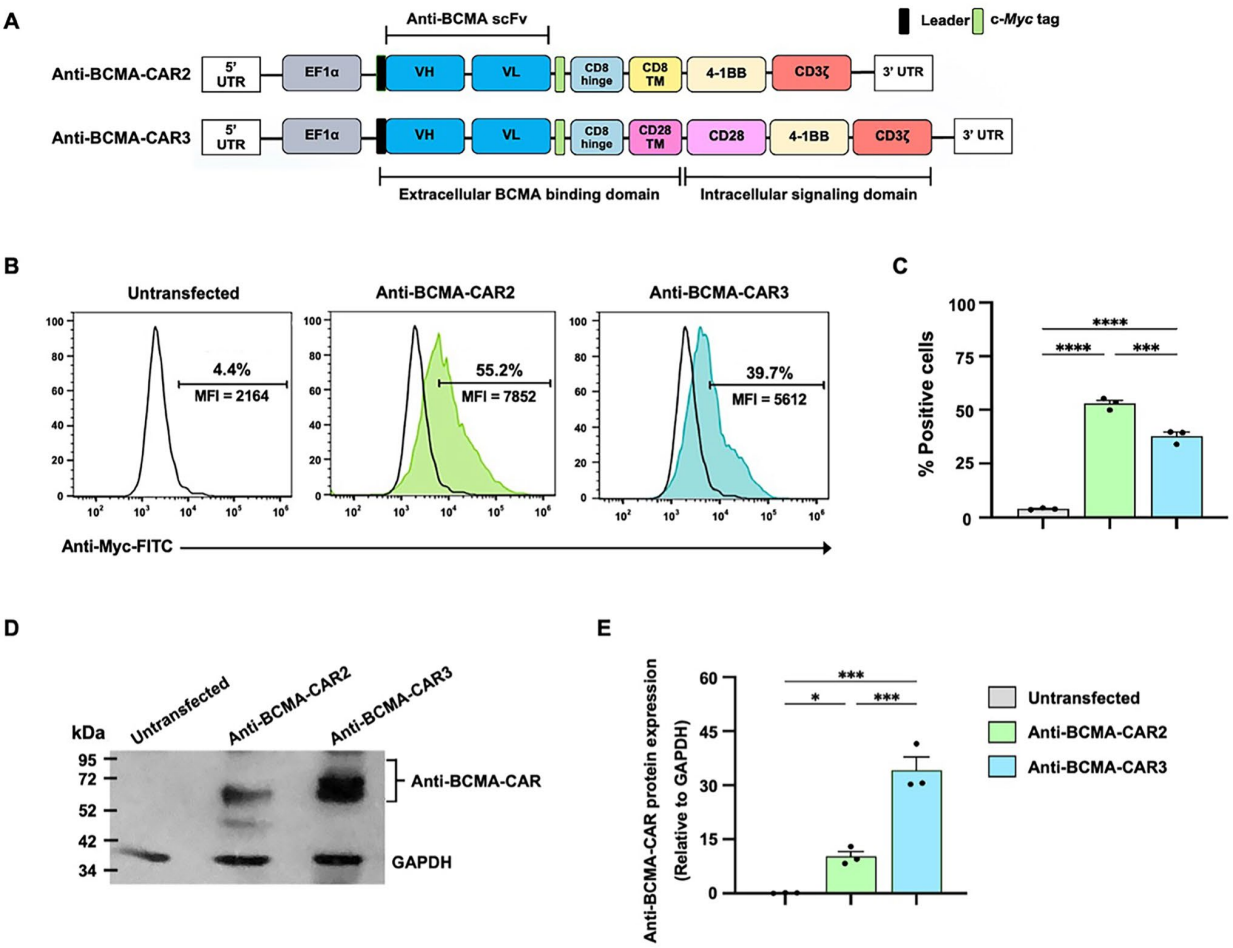
Lentiviral constructs of anti-BCMA-CARs and protein expression in Lenti-X™ HEK293T cells. **A** Schematic representation shows second-generation anti-BCMA-CAR (anti-BCMA-CAR2) and third-generation anti-BCMA-CAR (anti-BCMA-CAR3) lentiviral constructs containing fully human anti-BCMA scFv, c-Myc tag, hinge region, transmembrane (TM) domain, co-stimulatory domain(s), and CD3ζ. **B** Representative histogram demonstrates the expression of anti-BCMA-CARs on the surface of Lenti-X™ HEK293T cells analyzed by flow cytometry using by anti-cMyc-FITC antibody, and **C** the data were summarized as bar graphs. **D** Immunoblot analysis of cell lysates of Lenti-X™ HEK293T cells using anti-CD3ζ antibody shows specific bands of anti-BCMA-CAR2 and anti-BCMA-CAR3 at 62 and 66 kDa, respectively, with a loading control of 37 kDa GAPDH. **E** The densitometry data of anti-BCMA-CAR protein bands relative to GAPDH bands on SDS-PAGE analyzed using ImageJ software were summarized as bar graphs. The data obtained from 3 independent experiments were expressed as mean ± standard error of the mean (SEM) (N = 3). Statistical significance was determined using one-way analysis of variance (ANOVA) with Tukey’s post-hoc test (*p < 0.05, **p < 0.01, ***p < 0.001, and ****p < 0.0001)

The low complete response (CR) rate of CAR T cells, which may be due to decreased efficacy of murine single-chain variable fragment (scFv) anti-BCMA chimeric antigen receptor (CAR) T cells, has motivated efforts to enhance anti-BCMA-CAR T functions. In this regard, we generated two generations of fully human scFv anti-BCMA-CARs: anti-BCMA-CAR3 (CD28/4-1BB/CD3ζ) and anti-BCMA-CAR2 (4-1BB/CD3ζ), the latter of which was generated for comparison purposes (Fig. 2A). Both CARs were produced using the EF1α promoter. The fully human scFv sequence targeting BCMA was obtained from the international publication WO 2016/014565 A2 with BCMA-10 (EG63-98LB; 139109). A c-Myc tag was inserted after the CAR sequence, followed by the CD8 hinge and CD28 or CD8 transmembrane domain (Fig. 2A).

To investigate the expression of anti-BCMA-CAR proteins in Lenti-X™ HEK293T cells before T cell transduction, lentiviral constructs containing anti-BCMA-CAR2 or anti-BCMA-CAR3 were transfected into these cells. Flow cytometric analysis revealed a significant increase in surface c-Myc expression on anti-BCMA-CAR2-transfected cells (52.9 ± 1.6%) and anti-BCMA-CAR3-transfected cells (37.7 ± 1.9%) compared to un-transfected Lenti-X™ HEK293T (UTF) cells (4.0 ± 0.2%) (Fig. 2B, C). The expression of CD3ζ was assessed by immunoblotting, as shown in Fig. 2D, E. Specifically, the intracellular CD3ζ immunoblot results displayed a specific band of anti-BCMA-CAR2 and anti-BCMA-CAR3 at 62 and 66 kDa, respectively, with 37 kDa of GAPDH as a loading control (Fig. 2D). Furthermore, when analyzing the densitometry results of anti-BCMA-CAR protein bands relative to GAPDH bands on SDS-PAGE using ImageJ software, the results showed that anti-BCMA-CAR2 and anti-BCMA-CAR3 had significantly different protein expression from un-transfected Lenti-X™ HEK293T cells with *p* = 0.0465 and *p* = 0.0001, respectively. Notably, anti-BCMA-CAR3 demonstrated a significantly higher expression of anti-BCMA-CAR protein than anti-BCMA-CAR2 with *p* = 0.0008 (Fig. 2E). The findings of this study suggest that in the mammalian cell system, both the anti-BCMA-CAR2 and anti-BCMA-CAR3 constructs expressed CAR protein containing CD3ζ.

### Generation and characterization of anti-BCMA-CAR2 and anti-BCMA-CAR3 T cells

**Fig. 3:**
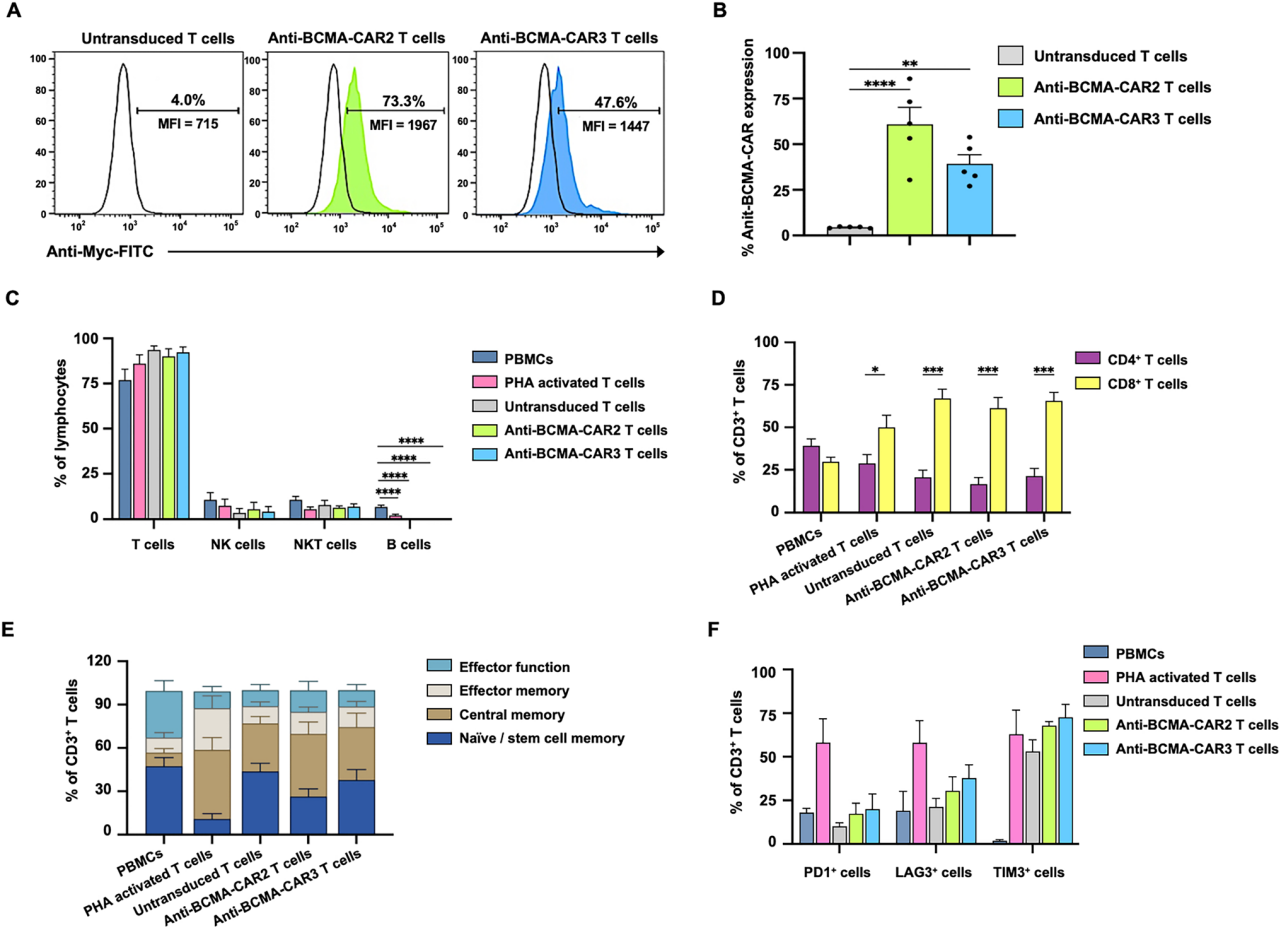
Generation and characterization of anti-BCMA-CAR T cells. **A** Histogram plots were used to demonstrate the transduction efficiency of chimeric antigen receptor (CAR) constructs to express an anti-BCMA-CAR protein on T cells isolated from peripheral blood mononuclear cells (PBMCs) of healthy volunteer donors. The anti-c-Myc FITC antibody was employed for surface staining. **B** Surface expression of anti-BCMA-CAR was assessed in a sample size of N = 5. **C** The immune cell population was characterized based on CD3 expression (T cells), CD3^-^CD56^+^CD16^+^ (natural killer (NK) cells), CD3^+^CD56^+^ (natural killer T (NKT) cells), and CD3^-^CD19^+^ (B cells). **D** Helper (CD3^+^CD4^+^) and cytotoxic (CD3^+^CD8^+^) T-cell percentages. **E** Flow cytometric analysis was used to investigate the expression of T cell subsets on CD3^+^ lymphocytes. **F** Three exhaustion markers, PD-1, LAG-3, and TIM-3 were evaluated in CD3^-^ cells using flow cytometry. Data from 5 individual healthy donors are presented as mean ± standard error of the mean (SEM) (N = 5). Unpaired Student’s t-tests were used to compare two groups, and one-way ANOVA with Tukey’s post-hoc test was employed to determine statistical significance (*p < 0.05, **p < 0.01, ***p < 0.001, and ****p < 0.0001)

The present study describes the generation of anti-BCMA-CAR2 and anti-BCMA-CAR3 T cells via lentiviral transduction of primary human T cells with lentiviral particles carrying genes encoding the respective CARs. Transduction efficiencies were assessed by measuring anti-BCMA-CAR surface expression on T cells, which showed that 4.5 ± 0.2% of untransduced T cells (UTD T) expressed the CAR, while 60.8 ± 9.4% and 39.2 ± 5.0% of anti-BCMA-CAR2 and anti-BCMA-CAR3 T cells, respectively, expressed the CAR (Fig. 3A, B). The immunophenotypes of anti-BCMA-CAR T cells were characterized, revealing that more than 90% of the T cell population expressed CD3 (Fig. 3C), while the populations of NK and NKT cells were low and not significantly different among the experimental groups. Interestingly, the population of B cells in PBMCs was reduced in PHA-L activated-T cells, UTD T cells, and anti-BCMA-CAR T cells (Fig. 3C). Further analysis of the CD3 + population showed that cytotoxic T cells (CD8^+^) were significantly more prevalent than helper T cells (CD4^+^) in PHA-L activated-T cells (50 ± 7.3% and 28.7 ± 5.2%), UTD T cells (67 ± 5.6% and 20.6 ± 4.3%), anti-BCMA-CAR2 T cells (61.3 ± 6.3% and 16.8 ± 3.8%), and anti-BCMA-CAR3 T cells (65.5 ± 5.1% and 21.3 ± 4.5%) (Fig. 3D). Analysis of the T cell subsets, including naive/stem cell memory, central memory, effector memory, and effector function cells, showed no significant difference between anti-BCMA-CAR T cells and UTD T cells (Fig. 3E). Finally, PD-1 and LAG-3 expression was higher in PHA-L activated-T cells compared to PBMCs, UTD T cells, anti-BCMA-CAR2, and anti-BCMA-CAR3 T cells (Fig. 3F). However, the expression of TIM-3 was increased in all groups, including PHA-L activated-T cells, UTD T cells, anti-BCMA-CAR2 T cells, and anti-BCMA-CAR3 T cells, compared to PBMCs (1.8 ± 0.7%) (Fig. 3F).

### Anti-tumor activities of anti-BCMA-CAR2 and anti-BCMA-CAR3 T cells against multiple myeloma cells expressing BCMA

**Fig. 4:**
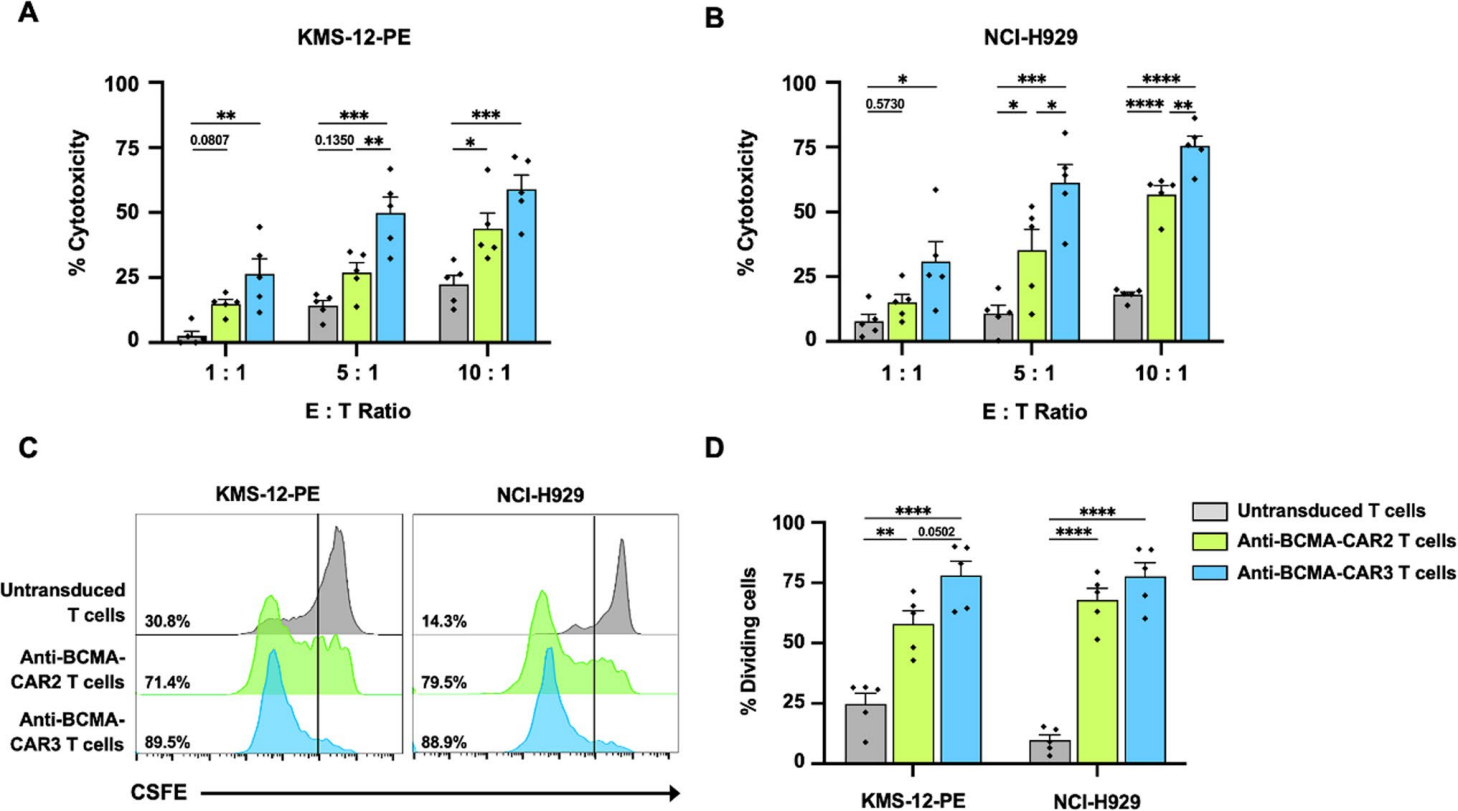
The anti-tumor effect and proliferation activity of anti-BCMA-CAR T cells against multiple myeloma (MM) expressing B cell maturation antigen (BCMA). The killing activities of untransduced (UTD) T cells, anti-BCMA-CAR2 T cells, and anti-BCMA-CAR3 T cells against **A** KMS-12-PE (BCMA^low^), and **B** NCI-H929 (BCMA^high^) cells were evaluated at different effector to target (E:T) ratios of 1:1, 5:1, and 10:1 over a 12-h co-culture period. The number of viable target cells was then determined using a counting bead and analyzed via flow cytometry. **C** The histogram and **D** the percentage of cell proliferation of UTD T cells, anti-BCMA-CAR2 T cells, and anti-BCMA-CAR3 T cells after activation by co-culturing with KMS-12-PE (BCMA^low^) cells and NCI-H929 (BCMA^high^) at an effector to target (E:T) ratio of 5:1 for five days in the absence of exogenous cytokines were examined by carboxyfluorescein diacetate succinimidyl ester (CFSE) dilution through flow cytometry. The data were collected from 5 individual healthy donors and presented as mean values with standard error of the mean (SEM) (N = 5). One-way ANOVA with Tukey’s post-hoc test was performed to evaluate the statistical significance of the results, denoted as *p < 0.05, **p < 0.01, ***p < 0.001, and ****p < 0.0001

This experiment was conducted to investigate the anti-tumor activities of anti-BCMA-CAR2 and anti-BCMA-CAR3 T against BCMA-expressing multiple myeloma (MM) cell lines. K562 cells were utilized as negative target cells (BCMA^neg^), whereas KMS-12-PE (BCMA^low^) and NCI-H929 (BCMA^high^) cells were employed as MM target cells that express BCMA. Target cells were cocultured with either untransduced (UTD) T cells or anti-BCMA-CAR T cells at various effector-to-target (E:T) ratios (1:1, 5:1, and 10:1) for a duration of 12 h. After coculture, the viability of target cells was evaluated using flow cytometry. The outcomes indicated that both anti-BCMA-CAR2 and anti-BCMA-CAR3 T cells displayed minimal cytotoxicity against K562 (BCMA^neg^) cells in comparison to control UTD T cells (Supplementary Fig. 1). However, anti-BCMA-CAR2 and anti-BCMA-CAR3 T cells exhibited specific dose-dependent killing of BCMA-expressing MM cells, KMS-12-PE, and NCI-H929 cells (Fig. 4A, B). The cytotoxicity of anti-BCMA-CAR2 and anti-BCMA-CAR3 T cells against KMS-12-PE cells was observed to be up to 43.8 ± 6.1% and 59 ± 5.4%, respectively, at an E:T ratio of 10:1 (Fig. 4A). The percentage of cytotoxicity against NCI-H929 cells by anti-BCMA-CAR2 and anti-BCMA-CAR3 T cells was noted to be up to 56.7 ± 3.4% and 75.5 ± 3.8%, respectively, at an E:T ratio of 10:1 (Fig. 4B).

### Stimulation of anti-BCMA-CAR2 and anti-BCMA-CAR3 T cell proliferation through co-culture with BCMA-expressing multiple myeloma cells

Upon co-culturing with KMS-12-PE (BCMA^low^) or NCI-H929 (BCMA^high^) cells at the effector to target (E:T) ratio of 5:1, we examined the proliferation of UTD, anti-BCMA-CAR2, and anti-BCMA-CAR3 T cells by analyzing the dilution of carboxyfluorescein succinimidyl ester (CFSE) in proliferating T cells on day 5. Our results indicate that after co-culturing with KMS-12-PE (BCMA^low^) cells, anti-BCMA-CAR2 T cells proliferated significantly higher than UTD T cells (58.0 ± 5.4% and 24.9 ± 4.4%, respectively; *p* = 0.0022). Interestingly, after co-culturing with KMS-12-PE (BCMA^low^) cells, anti-BCMA-CAR3 T cells proliferated even greater than UTD T cells (78.0 ± 6.0% and 24.9 ± 4.4%, respectively; *p* < 0.0001) (Fig. 4C, D). We observed similar results when co-culturing with NCI-H929 (BCMA^high^) cells, where anti-BCMA-CAR2 T cells proliferated significantly higher than UTD T cells (67.9 ± 4.9% and 9.8 ± 2.3%, respectively; *p* < 0.0001). Additionally, anti-BCMA-CAR3 T cells proliferation was significantly higher than UTD T cells (77.7 ± 5.6% and 9.8 ± 2.3%, respectively; *p* < 0.0001) upon co-culturing with NCI-H929 (BCMA^high^) cells (Fig. 4C, D).

### Cytokine production of anti-BCMA-CAR2 and anti-BCMA-CAR3 T cells in response to multiple myeloma cells expressing BCMA

**Fig. 5:**
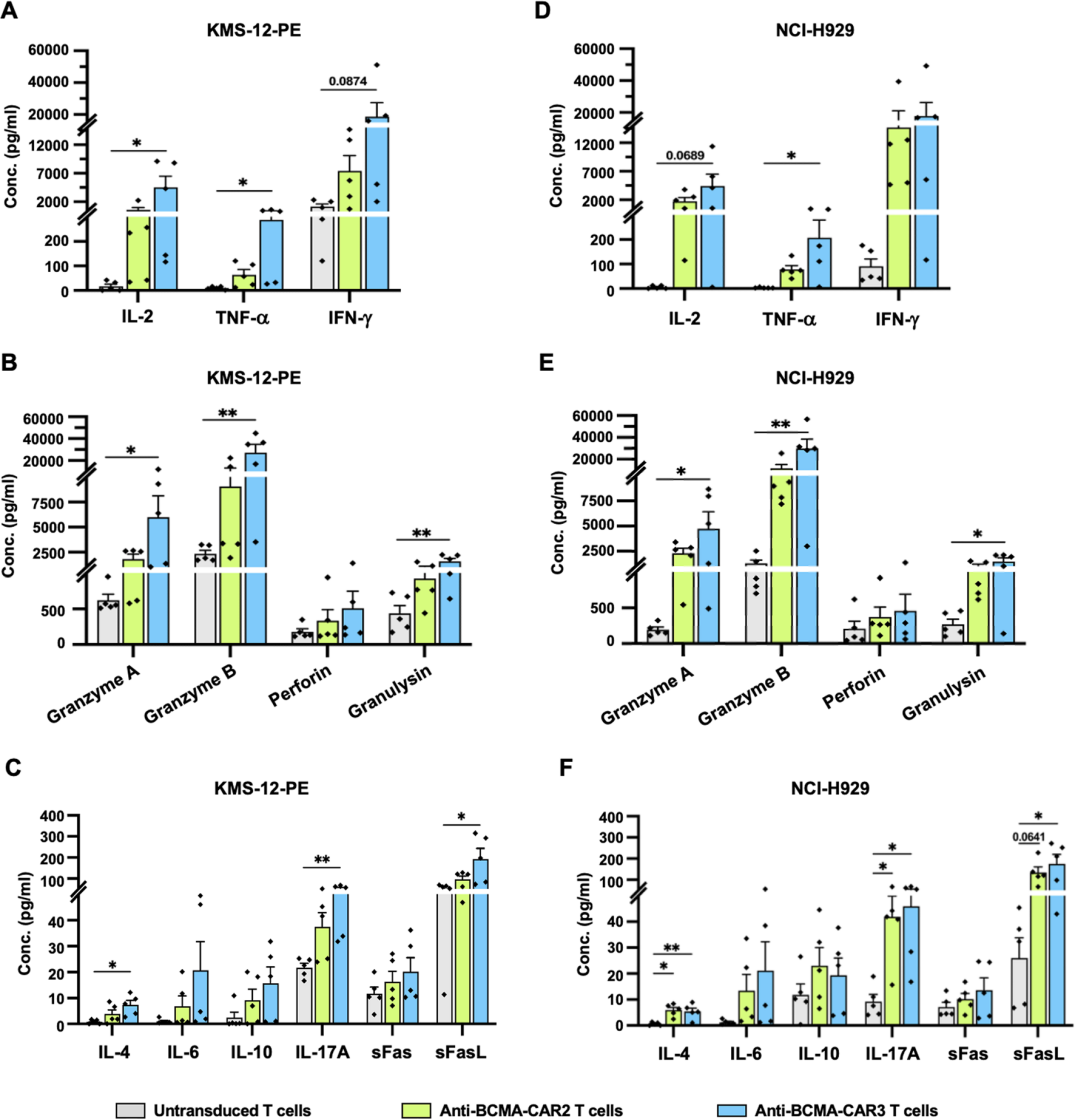
Cytokine production levels of anti-BCMA-CAR T cells against multiple myeloma (MM) cell lines expressing B-cell maturation antigen (BCMA). The levels of IL-2, TNF-α, IFN-γ, (**A**,** D**) Granzyme-A, Granzyme-B, Perforin, Granulysin (**B**,** E**), as well as IL-4, IL-6, IL-10, IL-17A, sFas, and sFasL (**C**,** F**) in the cell culture supernatants of anti-BCMA-CAR T cells, were analyzed using cytometric bead array (CBA) after 24 h of activation by culturing with KMS-12-PE (BCMA^low^) cells and NCI-H929 (BCMA^high^) cells at an E:T ratio of 5:1. The data were collected from 5 individual healthy donors and presented as mean ± standard error of the mean (SEM) (N = 5). One-way ANOVA with Tukey’s post-hoc test was utilized to determine statistical significance (*p < 0.05, **p < 0.01, ***p < 0.001, and ***p < 0.0001)

In this study, we investigated cytokine production in response to multiple myeloma (MM) cells expressing BCMA using KMS-12-PE (BCMA^low^) or NCI-H929 (BCMA^high^) cells co-cultured with UTD, anti-BCMA-CAR2, or anti-BCMA-CAR3 T cells at an effector to target (E:T) ratio of 5:1. Cytokine levels were measured by LEGENDplex™ Human CD8/NK cell panel Cytokine Bead Array (CBA) of 13 cytokines and proteins after 24 h of co-culture. Online Resource shows the cytokine concentration of anti-BCMA-CAR2 and anti-BCMA-CAR3 T cells against KMS-12-PE and NCI-H929 cells compared to UTD T cells. The data demonstrated that the levels of IL-2 and TNF-α in the culture media of anti-BCMA-CAR3 T cells co-cultured with KMS-12-PE (BCMA^low^) cells were significantly increased (561.6 ± 422.3 pg/ml and 288.2 ± 117.6 pg/ml, respectively) compared to UTD T cells (16.8 ± 8.7 pg/ml and 9.8 ± 2.8 pg/ml, respectively) (Fig. 5A). However, there was no significant difference in IFN-γ levels. Additionally, the levels of granzyme A, granzyme B, and granulysin were significantly increased in the culture media of anti-BCMA-CAR3 T cells co-cultured with KMS-12-PE (BCMA^low^) cells (5,995.8 ± 2,141.8 pg/ml, 27,248 ± 7,496.4 pg/ml, and 1,579.4 ± 323.8 pg/ml, respectively), compared to the UTD T cells (623.5 ± 82.6 pg/ml, 2352.7 ± 355.5 pg/ml, and 436.7 ± 112.9 pg/ml, respectively) (Fig. 5B). Furthermore, the levels of IL-4, IL-17A, and sFasL were significantly increased in the culture supernatant of anti-BCMA-CAR3 T cells against KMS-12-PE (BCMA^low^) cells (7.3 ± 1.8 pg/ml, 50.7 ± 7.5 pg/ml, and 192.3 ± 50.8 pg/ml, respectively), compared to UTD T cells (0.8 ± 0.4 pg/ml, 21.7 ± 1.6 pg/ml, and 51.7 ± 10.5 pg/ml, respectively) (Fig. 5C). These findings suggest that anti-BCMA-CAR3 T cells can produce cytokines and proteins in response to BCMA-expressing MM cells, which could potentially contribute to an anti-tumor immune response.

The present study also examined the cytokine production of anti-BCMA-CAR2 and anti-BCMA-CAR3 T cells during co-culture with NCI-H929 (BCMA^high^) cells. The result demonstrated that the level of TNF-α was significantly elevated in the culture media of anti-BCMA-CAR3 T cells (206.4 ± 72.9 pg/ml) compared to UTD T cells (2.8 ± 0.8 pg/ml) (Fig. 5D). Furthermore, the levels of granzyme A, granzyme B, and granulysin were significantly increased in the culture media of anti-BCMA-CAR3 T cells co-cultured with NCI-H929 cells (BCMA^high^) cells (4,727.8 ± 1,679.1 pg/ml, 29,991 ± 8,511.2 pg/ml, and 14,62.4 ± 388.7 pg/ml, respectively) relative to UTD T cells (193.4 ± 37.2 pg/ml, 1,278.3 ± 334.4 pg/ml, and 270 ± 72.9 pg/ml, respectively) (Fig. 5E). Moreover, the levels of IL-4 were significantly increased in both anti-BCMA-CAR2 and anti-BCMA-CAR3 T cells (5.9 ± 1.1 pg/ml and 5.4 ± 1.3 pg/ml, respectively) relative to UTD T cells (0.6 ± 0.3 pg/ml) (Fig. 5F). Similarly, the level of IL-17A was significantly elevated in both anti-BCMA-CAR2 and anti-BCMA-CAR3 T cells (41.8 ± 8.1 pg/ml and 45.9 ± 9.9 pg/ml, respectively) compared to UTD T cells (9.2 ± 2.7 pg/ml) (Fig. 5F). Additionally, the level of sFasL was significantly increased in the culture media of anti-BCMA-CAR3 T cells (174.9 ± 44.7 pg/ml) compared to UTD T cells (25.9 ± 7.8 pg/ml) (Fig. 5F).

### Anti-tumor efficiency of anti-BCMA-CAR2 and anti-BCMA-CAR3 T cells against multiple myeloma cells expressing BCMA in a long-term treatment

**Fig. 6:**
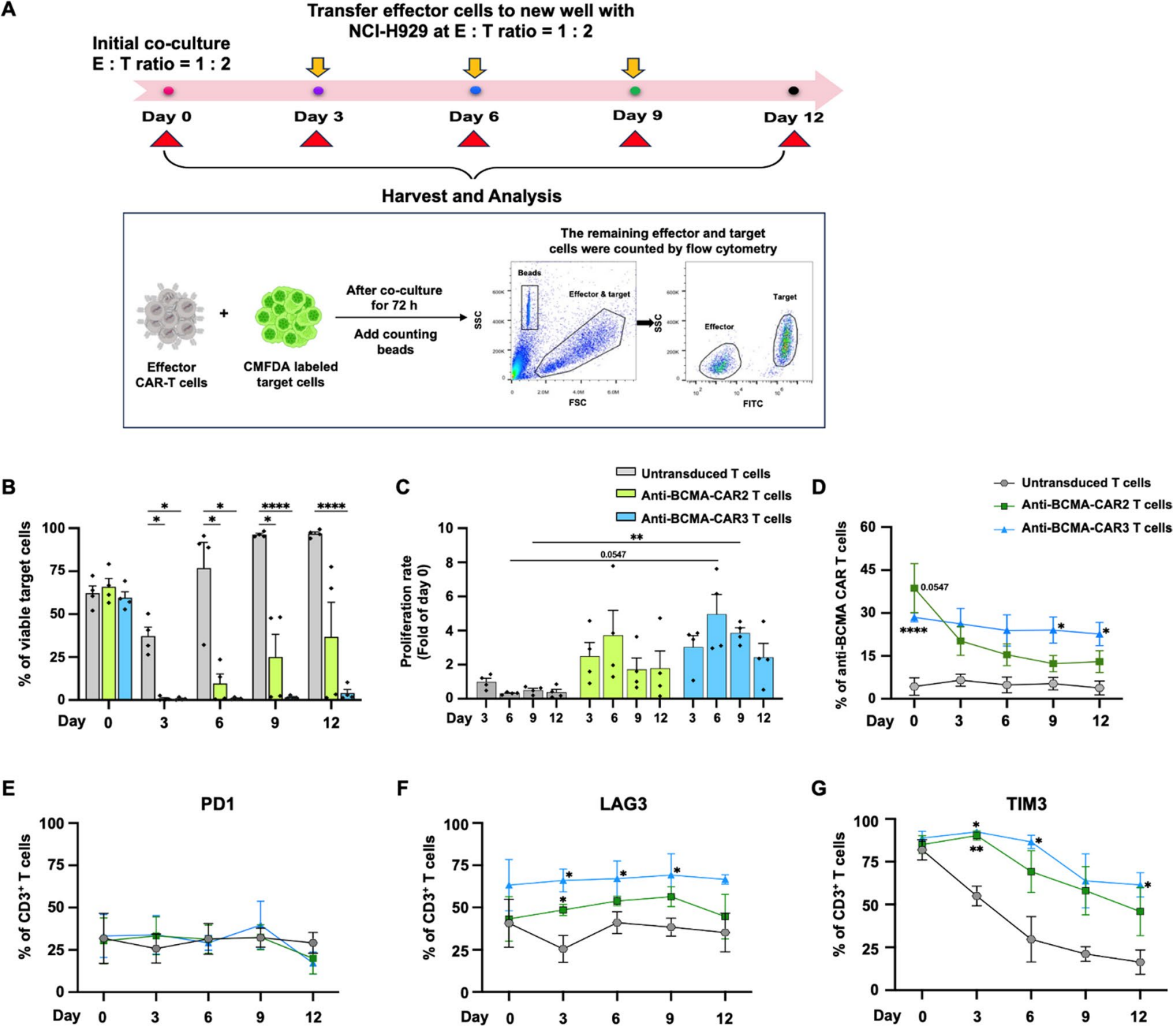
The efficiency of anti-BCMA-CAR T cells against BCMA expressed on multiple myeloma over an extended period. **A** The schematic overview of the stress test experiment illustrates the co-culture of anti-BCMA-CAR T cells with NCI-H929 target cells, subjected to antigenic stimulation for three cycles at an effector (E) to target (T) ratio of 1:2. Over the 12-day period, the following parameters were assessed: **B** residual viable BCMA target cells, **C** proliferation rate, **D** frequency of transduced CAR^+^, and the expression of exhaustion markers, including **E** PD-1, **F** LAG-3, and **G** TIM-3. The data collected from 4 individual healthy donors are presented as the mean ± standard error of the mean (SEM) (N = 4). Two-way ANOVA was used to determine statistical significance (*p < 0.05, **p < 0.01, ***p < 0.001, and ***p < 0.0001)

In order to evaluate anti-tumor efficiency in long-term treatment, anti-BCMA-CAR2 or anti-BCMA-CAR3 T cells were co-cultured with NCI-H929 (BCMA^high^) cells at E:T ratio of 1:2. The target MM cells were repeatedly added to the CAR T cells at days 3, 6, 9, and 12, and the residual viable NCI-H929 (BCMA^high^) cells were collected to investigate cell viability (Fig. 6A). The co-culturing with UTD T cells resulted in an increased number of tumor cells on days 6, 9, and 12, with percentages of 76.9 ± 15%, 96.3 ± 0.7%, and 96.9 ± 1.1%, respectively. However, anti-BCMA-CAR2 T cells were able to significantly reduce the number of target cells at days 3, 6, and 9 (0.9 ± 0.6%, 9.7 ± 5.5%, and 25.1 ± 13.2%, respectively), with the number gradually increasing by day 12 (36.8 ± 20.1%) (Fig. 6B). Remarkably, anti-BCMA-CAR3 T cells almost completely eliminated the target cells and were significantly different from UTD T cells on days 3, 6, 9, and 12, with the remaining target cells at 4.1 ± 2.1% (*p* < 0.0001) (Fig. 6B).

This study also investigated the proliferation of CAR T cells in response to co-culturing with target MM cells expressing BCMA. The absolute effector cell counts were analyzed using a bead-based assay on days 3, 6, 9, and 12. The results demonstrated a significant increase in the proliferation rate of anti-BCMA-CAR3 T cells on day 9 (3.9 ± 0.3-fold, *p* = 0.0015) compared to the UTD T cells (0.5 ± 0.1-fold), while anti-BCMA-CAR2 T cells showed a higher but not statistically significant increase compared to UTD T cells (Fig. 6C). Additionally, CAR expression in CAR T cells was evaluated by flow cytometry analysis of CD3^+^ and Myc-tag^+^ cells. At day 0, CD3^+^ T cells expressing anti-BCMA-CAR2 and anti-BCMA-CAR3 were detected at 38.7 ± 8.6% and 28.5 ± 1.6% *p* < 0.001, respectively. Following co-culturing with target MM cells to day 9 and day 12, anti-BCMA-CAR3 T cells showed significantly higher populations (24.1 ± 4.6% and 22.6 ± 4.1%, respectively) compared to UTD T cells (*p* = 0.0483 and 0.0320), whereas anti-BCMA-CAR2 T cells did not (12.3 ± 2.9% and 13.0 ± 0.8%) (Fig. 6D).

In addition, we also investigated the expression of exhaustion markers (PD-1, LAG-3, and TIM-3) in CAR T cells following prolonged stimulation with target cells. Flow cytometry was used to analyze the expression of these markers. Our findings revealed no differences in PD-1 expression across all conditions (Fig. 6E). However, LAG-3 expression was significantly higher in anti-BCMA-CAR3 T cells on day 3 (66.1 ± 3.4%; *p* = 0.0205), day 6 (67.1 ± 5.2%; *p* = 0.0500), and day 9 (69.2 ± 6.3%; *p* = 0.0238) when compared to UTD T cells (25.5 ± 8.0%, 41.1 ± 6.5%, and 38.5 ± 5.3%, respectively) but did not differ significantly from anti-BCMA-CAR2 T cells (Fig. 6F). Similarly, TIM-3 expression was significantly higher in anti-BCMA-CAR2 T cells and anti-BCMA-CAR3 T cells at day 3 (90.3 ± 2.7% and 92.4 ± 1.1%, respectively) when compared to UTD T cells (55.1 ± 5.7 pg/ml). However, at day 12, the TIM-3 expression was decreased in all groups (anti-BCMA-CAR2 T cells, anti-BCMA-CAR3 T cells, and UTD T cells) (46 ± 14.2%, 61.5 ± 7.2%, and 16.4 ± 7.2%, respectively) (Fig. 6G).

## Discussion

Currently, two anti-BCMA-CAR T cell products, namely ide-cel and cilta-cel, have received clinical approval for treating relapsed/refractory multiple myeloma (RRMM), [12, 27], both of which are second-generation CARs (CAR2) that use the 4-1BB intracytoplasmic co-stimulatory domain. Anti-BCMA-CAR2 T cells have demonstrated significant improvement in overall response rate (ORR) and survival compared to standard regimens in diverse BCMA-directed CAR T trials [14, 17, 19, 28]. However, some studies have reported a median progression-free survival (PFS) of less than 1 year and a lack of plateau or type I curve in the survival curve, leading to patient relapses [14]. The mechanisms underlying resistance and relapse after CAR T cell therapy in multiple myeloma are poorly understood, but one possible factor is the murine scFv induced-human anti-mouse antibody (HAMA) response, which reduces CAR T cell persistence in patients [29, 30]. To address this limitation, studies have attempted to develop humanized and fully human scFv to reduce immunogenicity and improve CAR T cell persistence [29, 31–33]. Another strategy is to add co-stimulatory molecules, although CD28-containing CAR T cells have been associated with faster CAR T cell exhaustion and shorter response duration than CAR T cells containing the 4-1BB domain [34, 35]. To potentially augment the efficiency of CAR T cells, we generated third-generation anti-BCMA-CAR (anti-BCMA-CAR3) T cells containing CD28/4-1BB/CD3ζ signaling domains, which may overcome the limitations of second-generation anti-BCMA-CAR (anti-BCMA-CAR2) T cells containing the 4-1BB/CD3ζ signaling domains.

To test this hypothesis, we created anti-BCMA-CAR3 T cells and examined their anti-tumor functionality, comparing them to anti-BCMA-CAR2 T cells targeting MM cell lines. Firstly, we investigated the expression of BCMA in MM cell lines, KMS-12-PE and NCI-H929 cells, and the CML cell line, K562 cells. BCMA is expressed on the surface of plasma cells and is often overexpressed on malignant plasma cells as compared to normal plasma cells [36], making it an ideal target for immunotherapies in MM. Notably, two FDA-approved CAR T cell products for RRMM also target BCMA as the antigen [15]. We found that K562 cells lacked BCMA expression, whereas NCI-H929 cells exhibited high and KMS-12-PE cells showed moderate to low BCMA expression, respectively (Fig. 1). Furthermore, the immunoblot analysis of NCI-H929 revealed the presence of multiple bands (Fig. 1C). This observation is consistent with the findings of Huang et al. [37], who noted that BCMA in MM cells, especially within the NCI-H929 cell line, is characterized as an *N*-glycosylated protein. Thus, the occurrence of multiple bands in NCI-H929 can be attributed to this *N*-glycosylation phenomenon, as elucidated by Huang et al. As a result, we employed K562 cells as negative target cells (BCMA^neg^), while KMS-12-PE (BCMA^low^) and NCI-H929 (BCMA^high^) cells were utilized as MM target cells with varying levels of BCMA expression to assess the efficacy of CAR T cells.

Co-stimulatory molecules are known to modulate the anti-tumor activity, proliferation, cytokine production, and persistence of CAR T cells. In this investigation, we designed lentiviral vectors incorporating a fully human anti-BCMA scFv protein linked to a CAR cassette containing either 4-1BB/CD3ζ (anti-BCMA-CAR2) or CD28/4-1BB/CD3ζ (anti-BCMA-CAR3) co-stimulatory domains (Fig. 2A). CD28 is expressed in both resting and activated T cells, and has been demonstrated to promote T cell proliferation, IL-2 and Th1 cytokine production, as well as activation-induced cell death resistance of CAR T cells [38]. CAR T cells containing CD28 have been shown to enhance anti-cancer functions and persistence both in vitro and in vivo [39]. Moreover, it enhanced CAR T cell activity, expansion, and longer persistence in lymphoma patients [40]. Therefore, the addition of CD28 could potentially enhance the anti-tumor function, proliferation, and persistence of anti-BCMA-CAR3 T cells. The expression of anti-BCMA-CAR2 and anti-BCMA-CAR3 proteins was confirmed in Lenti-X™ HEK293T cells (Fig. 2B–D), and the T cells expressing these CARs were successfully generated from human primary T lymphocytes with only slight differences in CAR protein expression (Fig. 3A, B). The phenotypic analysis of cells showed a decrease in B cells (Fig. 3C) but an increase in cytotoxic T (CD3^+^/CD8^+^) cells (Fig. 3D), which may be attributed to the presence of IL-2, IL-7, and IL-15 cytokines in the culture conditions. Another study has demonstrated that antigen-specific CD8^+^T cells could be significantly expanded after antigen stimulation and the addition of IL-2 or IL-15 cytokines [41]. The anti-BCMA-CAR2 and anti-BCMA-CAR3 T cells, as well as PBMCs and UTD T cells, showed a decrease in exhaustion markers, PD-1, compared to PHA-activated T cells (Fig. 3F). This suggests that anti-BCMA-CAR3 did not significantly alter the phenotypes of T cells. Furthermore, the central memory T cell subtype (Tcm) was the predominant population in both anti-BCMA-CAR2 and anti-BCMA-CAR3 T cells (Fig. 3E). The increase in Tcm population might be associated with IL-7 and IL-15 cytokines in the culture system. A recent in vivo study has also demonstrated increased T-memory stem cell markers in the IL-7 and IL-15-preserved CAR.CD19 T cells [42]. These findings suggest that our production protocol facilitated the functionality of anti-BCMA-CAR2 and anti-BCMA-CAR3 T cells by maintaining cytotoxic T cells (CD3^+^/CD8^+^) and Tcm phenotypes, which may contribute to the persistence and cytotoxic function of CAR T cells [43, 44].

We then investigated the anti-tumor activity of anti-BCMA-CAR2 and anti-BCMA-CAR3 T cells against BCMA-expressing MM cells. To assess the specificity of anti-BCMA-CAR T cells, we compared their cytotoxicity against K562 (BCMA^neg^) cells to that of UTD T cells. The results showed that the cytotoxic activity of anti-BCMA-CAR2 T cells and anti-BCMA-CAR3 T cells against K562 cells were not statistically significant, indicating the specificity of anti-BCMA-CAR T cells. Elevating the effector cell dose led to a heightened percentage of cytotoxicity (Supplementary Fig. 1), potentially attributable to an HLA mismatch between the effector cells derived from healthy donors and the target K562 cells. This discrepancy might trigger the immune cells of the donor to attack the target cells [45, 46]. Interestingly, anti-BCMA-CAR3 T cells showed significantly greater efficiency in destroying KMS-12-PE (BCMA^low^) and NCI-H929 (BCMA^high^) target cells than anti-BCMA-CAR2 T cells, particularly at low E:T ratio (Fig. 4A, B). This may be due to differences in binding affinity and signaling of the co-stimulatory domain in the CAR constructs. The fully human BCMA-10 scFv (EG63-98LB; 139109) used in this study had a low binding affinity when it was bound to recombinant BCMA protein with an affinity of 33 nM [47]. It is theorized that lower affinity scFv in the CARs may result in lower cytotoxic activity and reactivity against cells with low antigen density [48]. This phenomenon may be attributed to the observed anti-tumor function of anti-BCMA-CAR2 T cells. Interestingly, low-affinity constructs have been associated with reduced levels of antigen loss and promoted CAR T cell proliferation [49]. Drent et al. [50] discovered that when CD28 and 4-1BB co-stimulation are combined with very low-affinity scFv, the CAR T cells exhibit improved clinical potential, superior proliferative capacity, preservation of a central memory phenotype, and significantly improved in vivo anti-tumor function while retaining their ability to discriminate target antigen density. This supports our results that anti-BCMA-CAR3 T cells significantly killed both KMS-12-PE (BCMA^low^) and NCI-H929 (BCMA^high^) target cells better than anti-BCMA-CAR2 T cells (Fig. 4A, B). Additionally, anti-BCMA-CAR3 T cells exhibited a trend toward higher proliferation, particularly when cocultured with the KMS-12-PE (BCMA^low^) cell line (Fig. 4C, D).

Upon recognizing tumor antigens, CAR T cells secrete pro-inflammatory cytokines, such as IL-2, IFN-γ, and TNF-α, to regulate cell growth, activation, and differentiation [51]. Compared to UTD T cells, the levels of cytokine secretion, including IL-2, IFN-γ, and TNF-α from anti-BCMA-CAR2 and anti-BCMA-CAR3 T cells were significantly and similarly increased (Fig. 5A, D), indicating their antigen-specific response. It is worth noting that, in comparison to the UTD T cells, only the anti-BCMA-CAR3 T cells exposed to either KMS-12-PE (BCMA^low^) or NCI-H929 (BCMA^high^) target cells demonstrated significantly elevated levels of TNF-α (Fig. 5A, D). CAR-T cells also employ granule-mediated apoptosis for tumor cell destruction. In this study, the levels of granzyme A, granzyme B, and granulysin secretion from anti-BCMA-CAR2 and anti-BCMA-CAR3 T cells were markedly elevated compared to those of UTD T cells (Fig. 5B, E). Moreover, we observed that anti-BCMA-CAR3 T cells elicited higher levels of IL-4, IL-17A, and soluble Fas ligand (sFasL) release than UTD T cells after co-culture with KMS-12-PE (BCMA^low^) or NCI-H929 (BCMA^high^) cells (Fig. 5C, F). IL-17A exerts specific control over tissue infections via synergistic expression of pro-inflammatory cytokines and chemokines. On the other hand, IL-4, known as the “master immune-stimulating cytokine”, regulates antibody production, hematopoietic and inflammatory responses, and effector T cell responses [52, 53]. Additionally, CAR T cells can eliminate antigen-negative tumor cells within the antigen-positive environment via the Fas and Fas ligand (FasL) pathway [54]. The elevated sFasL in the co-culture conditions of anti-BCMA-CAR3 T cells, in comparison to UTD T cells, may further enhance their cytotoxic function. However, only the levels of IL-4 and IL-17A were significantly higher in anti-BCMA-CAR2 T cells than UTD T cells when co-cultured with NCI-H929 (BCMA^high^) cells. These results align with earlier studies indicating that CAR3 T cells secrete greater and more diverse cytokines than CAR2 T cells [23, 50] without triggering cytokine release syndrome (CRS) found in CAR2 T cells. Notably, a clinical investigation by Ramos  *et al*. (20) demonstrated that CAR3 T cells against CD19 (CD28 and 41-BB) exhibited stronger anti-tumor activity than CAR2 T cells (CD28 only), with mild CRS, in 16 patients with relapsed/refractory non-Hodgkin lymphoma.

The inability of CAR T cells to maintain their function over the long term has been proposed as a potential mechanism underlying disease progression [55]. In particular, chronic exposure to antigens, especially in the tumor microenvironment, may result in the loss of CAR T cell proliferation, cytokine production, and cytotoxicity, which can lead to disease relapse in certain patients, including those with MM [12]. Moreover, when CAR T cells lose their functionality, they may express immune checkpoint molecules such as PD-1, TIM-3, and LAG-3, which may further impede their anti-tumor activity [12, 27]. To test this hypothesis, we conducted an in vitro experiment using repeated serial stimulation to mimic chronic antigen exposure. Specifically, anti-BCMA-CAR T cells were harvested every 72 h and transferred to wells containing fresh NCI-H929 cells to maintain the E:T ratio at 1:2. This process was repeated four times, and the exhaustion and functional parameters of the CAR T cells were measured. Our results showed that repeated stimulation of anti-BCMA-CAR3 T cells with NCI-H929 (BCMA^high^) cells did not lose anti-tumor activity (Fig. 6B), indicating their excellent persistence. Furthermore, the preserved proliferative capacity of these CAR T cells (Fig. 6C, D) suggests that they are well-suited for long-term use. Although the expression of exhaustion markers, including PD-1, LAG-3, and TIM-3, in anti-BCMA-CAR3 T cells changed slightly during the observation period (Fig. 6E, F, G), this antigen-repeated stimulation did not lead to exhaustion of the anti-BCMA-CAR3 T cells. These findings are consistent with Zhang et al.[23] studies that demonstrated similar levels of exhaustion between CAR2 T cells and CAR3 T cells. Nevertheless, CAR3 T cells exhibited better proliferation and long-term persistence, resulting in more robust anti-tumor effects than CAR2 T cells [20, 21, 56].

Our study highlights the heightened anti-tumor efficacy of anti-BCMA-CAR3 T cells against BCMA-expressing MM cell lines in vitro when compared to anti-BCMA-CAR2 T cells. It is essential to note that our comparison focused on anti-BCMA-CAR3 T cells (CD28/4-1BB/CD3ζ) and anti-BCMA-CAR2 T cells (4-1BB/CD3ζ), and did not extend to a comparison between anti-BCMA-CAR3 T cells (CD28/4-1BB/CD3ζ) and anti-BCMA-CAR2 T cells (CD28/CD3ζ). The observed enhanced performance may be attributed to the interaction of CD28 and 4-1BB. Notably, studies by Ramos et al. [20], Zhang et al. [23], and Drent et al. [50] suggest that, when comparing third-generation CAR T cells (CD28/4-1BB/CD3ζ) with second-generation CAR T cells (CD28/CD3ζ), the former consistently demonstrates superior performance across different target antigens. Therefore, the outcomes of our study support the notion that third-generation CAR T cells (CD28/4-1BB/CD3ζ) outperform second-generation CAR T cells (4-1BB/CD3ζ), likely due the collaborative effects of CD28 and 41BB. Moreover, a comprehensive exploration necessitates pending in vivo investigations utilizing MM cancer xenograft mouse models. Furthermore, there exists potential for refining the anti-BCMA-CAR3 construct by synergistically incorporating additional costimulatory molecules, such as CD27, acknowledged for augmenting CAR T cell persistence [57]. Notably, engineered anti-CD19 CAR T cells that secrete anti-programmed death-ligand 1 (PD-L1) scFv demonstrate superior proliferation and enhanced anti-tumor functionality in comparison to anti-CD19 CAR T cells [58]. Hence, devising anti-BCMA-CAR3 T cells capable of secreting anti-PD-L1 scFv presents a promising avenue for enhancing their efficacy.

## Conclusion

In this investigation, we present a novel advancement: the development of third-generation CAR T cells targeting BCMA (anti-BCMA-CAR3 T cells). These CAR T cells incorporate a fully human single-chain variable fragment (scFv) against BCMA, along with two co-stimulatory molecules, CD28 and 4-1BB, linked to CD3ζ. Our analysis encompassed an assessment of the anti-tumor efficacy of these anti-BCMA-CAR3 T cells against MM cell lines expressing BCMA. This evaluation was juxtaposed with the performance of anti-BCMA-CAR2 T cells. Our findings illuminate that the anti-BCMA-CAR3 T cells can elicit targeted anti-tumor responses in MM cells expressing varying levels of BCMA, whether high or low. Remarkably, these cells demonstrated prolonged cellular proliferation and consistent expression of the CAR protein, exhibiting resilience against exhaustion even upon repetitive antigen exposure. Given these outcomes, the anti-BCMA-CAR3 T cells introduced in this inquiry present a highly promising avenue as a potential therapeutic modality. Consequently, they merit further advancement and exploration for their applicability in addressing BCMA-positive MM.

## Supplementary Information

Below is the link to the electronic supplementary material.

Supplementary file1 (DOCX 113 KB)

## Data Availability

Data are included in the manuscript.
