# Supplementary material for: Therapeutic potential of third-generation chimeric antigen receptor T cells targeting B cell maturation antigen for treating multiple myeloma
Source: Clin Exp Med. 2024 Apr 29;24(1):90. doi: 10.1007/s10238-024-01347-7 (PMC11058938; doi:10.1007/s10238-024-01347-7)
Supplement: Supplementary file 1 — Supplementary file1 (DOCX 113 KB) [file 10238_2024_1347_MOESM1_ESM.docx]

**Therapeutic Potential of Third-Generation Chimeric Antigen Receptor T Cells Targeting B Cell Maturation Antigen for Treating Multiple Myeloma**

Punchita Rujirachaivej^1#^, Teerapong Siriboonpiputtana^2#,^ Piriya Luangwattananun^3,4^, Pornpimon Yuti^3,4^, Yupanun Wutti-in^5^, Kornkan Choomee^3,4^, Jatuporn Sujjitjoon^3,4^, Takol Chareonsirisuthigul^2^, Budsaba Rerkamnuaychoke^2^, Mutita Junking^3,4*^ Pa-thai Yenchitsomanus^3,4*^

^1^Graduate Program in Clinical Pathology, Department of Pathology, Faculty of Medicine Ramathibodi Hospital, Mahidol University, Bangkok, Thailand

^2^Department of Pathology, Faculty of Medicine Ramathibodi Hospital, Mahidol University, Bangkok, Thailand

^3^Siriraj Center of Research Excellence for Cancer Immunotherapy (SiCORE-CIT), Faculty of Medicine Siriraj Hospital, Mahidol University, Bangkok, Thailand

^4^Division of Molecular Medicine, Research Department, Faculty of Medicine Siriraj Hospital, Mahidol University, Bangkok, Thailand

^5^Division of Transfusion Science, Department of Medical Technology, Faculty of Associated Medical Sciences, Chiang Mai University, Chiang Mai, Thailand

# These authors contributed equally to this work

***Correspondence to:** Mutita Junking, ORCID ID: 0000-0002-8347-3000, Siriraj Center of Research Excellence for Cancer Immunotherapy (SiCORE-CIT) and Division of Molecular Medicine, Research Department, Faculty of Medicine Siriraj Hospital, Mahidol University, Bangkok 10700, Thailand

E-mail: mutita.jun@mahidol.ac.th

and

Pa-thai Yenchitsomanus, ORCID ID: 0000-0001-9779-5927, Siriraj Center of Research Excellence for Cancer Immunotherapy (SiCORE-CIT) and Division of Molecular Medicine, Research Department, Faculty of Medicine Siriraj Hospital, Mahidol University, Bangkok 10700, Thailand

E-mail: ptyench@gmail.com

**Supplementary Fig. 1**

**
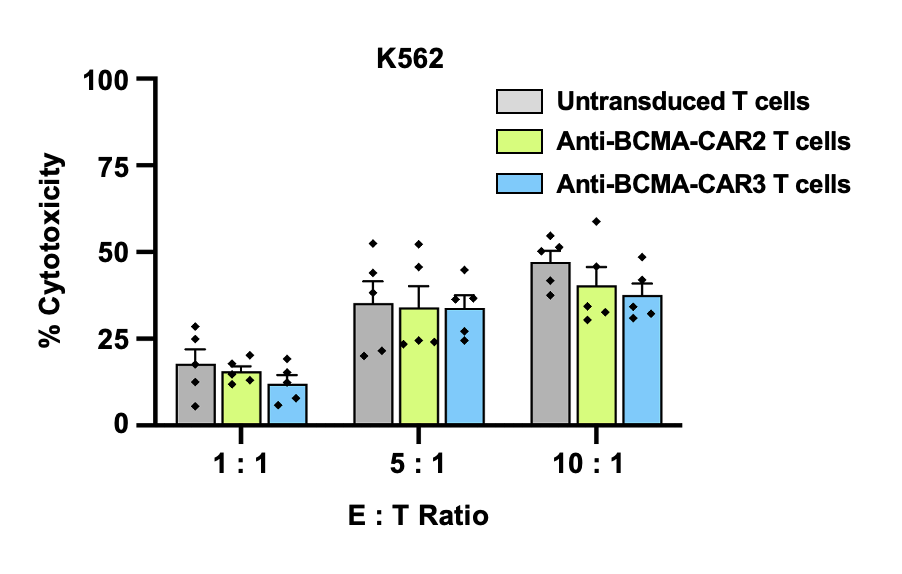
**

**Supplementary Fig. 1 A comparison of the anti-tumor effect of anti-BCMA-CAR T cells against multiple myeloma (MM) expressing B cell maturation antigen (BCMA).** The killing activities of untransduced (UTD) T cells, anti-BCMA-CAR2 T cells, and anti-BCMA-CAR3 T cells against K562 (BCMA^neg^) were evaluated at different effector to target (E:T) ratios of 1:1, 5:1, and 10:1 over a 12-hour co-culture period. The number of viable target cells was then determined using a counting bead and analyzed via flow cytometry. The data were collected from 5 individual healthy donors and presented as mean values with standard error of the mean (SEM) (N=5). One-way ANOVA with Tukey's post-hoc test was performed to evaluate the statistical significance of the results, denoted as **p*<0.05, ***p*<0.01, ****p*<0.001, and *****p*<0.0001

**Supplementary Table 1** **Cytokine production of untransduced T cells or anti-BCMA-CAR2 T cells or BCMA-CAR3 T cells, in response to the co-culturing with KMS-12-PE (BCMA^low^) cell line.** The cytokine levels were measured by LEGENDplex^TM^ Human CD8/NK cell panel Cytokine Bead Array (CBA) of 13 cytokines and proteins after 24 hours of the co-culture.

| **Cytokines** | **UTD T cells** | **anti-BCMA-CAR2 T cells** | | **anti-BCMA-CAR3 T cells** | |
| --- | --- | --- | --- | --- | --- |
|  | **Mean + SEM (pg/ml)** | **Mean + SEM (pg/ml)** | ***P**** | **Mean + SEM (pg/ml)** | ***P**** |
| **IL-2**  **IL-4 IL-6 IL-10 IL-17A**  **TNF-α**  **sFas**  **sFasL**  **IFN-γ**  **Granzyme A**  **Granzyme B**  **Perforin**  **Granulysin** | 16.8 ± 8.7  0.8 ± 0.4  1.0 ± 0.4  2.3 ± 2.1  21.7 ± 1.6  9.8 ± 2.8  11.6 ± 2.7  51.7 ± 10.5  1148.4 ± 446.1  623.5 ± 82.6  2352.7 ± 355.5  178.3 ± 43.4  436.7 ± 112.9 | 561.6 ± 422.3  3.8 ± 1.6  6.9 ± 3.9  9.2 ± 4.2  37.4 ± 5.5  63.7 ± 21.5  16.3 ± 4224.0  95.6 ± 17.1  7380.4 ± 2737.3  1815.6 ± 497.2  9060.3 ± 3998.3  335.9 ± 156.8  927.2 ± 185.8 | 0.9421  0.3207  0.8166  0.5550  0.1434  0.8473  0.7133  0.6002  0.6905  0.7884  0.6110  0.7892  0.3078 | 4510.4 ± 1979.6  7.3 ± 1.8  20.6 ± 11.1  15.6 ± 6.4  50.7 ± 7.5  288.2 ± 117.6  20.1 ± 5.5  192.3 ± 50.8  18693.4 ± 8732  5995.8 ± 2141.8  27248 ± 7496.4  509.8 ± 241.7  1579.4 ± 323.8 | 0.0457  0.0169  0.1451  0.1450  0.0070  0.0362  0.3613  0.0210  0.0874  0.0282  0.0097  0.3748  0.0096 |

* The data were obtained from 5 individual healthy donors, and the results are shown as mean ± standard error of the mean (SEM) (N=5). One-way analysis of variance (ANOVA) was used to determine statistical significance (N=5, **p*<0.05, ***p*<0.01, ****p*<0.001, and *****p*<0.0001).

**Supplementary Table 2 Cytokine production of untransduced T cells or anti-BCMA-CAR2 T cells or BCMA-CAR3 T cells, in response to the co-culturing with NCI-H929 (BCMA^high^) cell line.** The cytokine levels were measured by LEGENDplex^TM^ Human CD8/NK cell panel Cytokine Bead Array (CBA) of 13 cytokines and proteins after 24 hours of the co-culture.

| **Cytokines** | **UTD T cells** | **anti-BCMA-CAR2 T cells** | | **anti-BCMA-CAR3 T cells** | |
| --- | --- | --- | --- | --- | --- |
|  | **Mean + SEM (pg/ml)** | **Mean + SEM (pg/ml)** | ***P**** | **Mean + SEM (pg/ml)** | ***P**** |
| **IL-2**  **IL-4 IL-6 IL-10 IL-17A**  **TNF-α**  **sFas**  **sFasL**  **IFN-γ**  **Granzyme A**  **Granzyme B**  **Perforin**  **Granulysin** | 5.2 ± 2.9  0.6 ± 0.3  1.3 ± 0.4  11.8 ± 4.2  9.2 ± 2.7  2.8 ± 0.8  7.1 ± 1.8  25.9 ± 7.8  90.4 ± 0.4  193.4 ± 37.2  1278.3 ± 334.4  207 ± 108.6  270 ± 72.9 | 1748.4 ± 669.2  5.9 ± 1.1  13.5 ± 6.2  23.1 ± 7.0  41.8 ± 8.1  78.4 ± 15.4  10.2 ± 2.2  134 ± 26.3  14660 ± 6399.4  2302 ± 489.9  11778.2 ± 3451  369.8 ± 141.1  1017.9 ± 185.4 | 0.6015  0.0057  0.4940  0.4163  0.0250  0.4524  0.7774  0.0641  0.2542  0.3360  0.3719  0.7792  0.1322 | 4415.1 ± 2068.5  5.4 ± 1.3  21.1 ± 11.2  19.4 ± 6.6  45.9 ± 9.9  206.4 ± 72.9  13.6 ± 4.8  174.9 ± 44.7  17775.9 ± 8517.5  4727.8 ± 1679.1  29991 ± 8511.2  458.8 ± 232.9  1462.4 ± 388.7 | 0.0689  0.0112  0.1813  0.6610  0.0126  0.0149  0.3588  0.0117  0.1466  0.0203  0.0063  0.5603  0.0150 |

* The data were obtained from 5 individual healthy donors, and the results are shown as mean ± standard error of the mean (SEM) (N=5). One-way analysis of variance (ANOVA) was used to determine statistical significance (N=5, **p*<0.05, ***p*<0.01, ****p*<0.001, and *****p*<0.0001).
